# Association of age with healthcare needs and engagement among Nigerian men who have sex with men and transgender women: cross‐sectional and longitudinal analyses from an observational cohort

**DOI:** 10.1002/jia2.25599

**Published:** 2020-10-01

**Authors:** Habib O Ramadhani, Trevor A Crowell, Rebecca G Nowak, Nicaise Ndembi, Blessing O Kayode, Afoke Kokogho, Uchenna Ononaku, Elizabeth Shoyemi, Charles Ekeh, Sylvia Adebajo, Stefan D Baral, Manhattan E Charurat

**Affiliations:** ^1^ Institute of Human Virology University of Maryland School of Medicine Baltimore MD USA; ^2^ Henry M. Jackson Foundation for the Advancement of Military Medicine Inc Bethesda MD USA; ^3^ U.S. Military HIV Research Program Walter Reed Army Institute of Research Silver Spring MD USA; ^4^ Institute of Human Virology Nigeria Abuja Nigeria; ^5^ HJF Medical Research International Abuja Federal Capital Territory Nigeria; ^6^ Population Council Abuja Federal Capital Territory Nigeria; ^7^ Maryland Global Initiatives Corporation‐ A University of Maryland Baltimore Affiliate Abuja Nigeria; ^8^ Johns Hopkins School of Public Health Baltimore MD USA

**Keywords:** men who have sex with men, transgender people, HIV care continuum, STI, Delivery of Health Care, sexual and gender minorities, Africa South of the Sahara

## Abstract

**Introduction:**

Young men who have sex with men (MSM) and transgender women (TGW) face stigmas that hinder access to healthcare. The aim of the study was to understand age‐related determinants of healthcare needs and engagement among MSM and TGW.

**Methods:**

The TRUST/RV368 cohort provides integrated prevention and treatment services for HIV and other sexually transmitted infections (STIs) tailored to the needs of sexual and gender minorities. MSM and TGW aged ≥16 years in Abuja and ≥18 years Lagos, Nigeria, completed standardized behavioural questionnaires and were tested for HIV, *Neisseria gonorrhoeae* (NG) and *Chlamydia trachomatis* (CT) every three months for up to 18 months. Logistic regression was used to estimate adjusted odds ratios (aORs) for associations of age and other factors with outcomes of interest upon enrolment, including HIV care continuum steps – HIV testing, ART initiation and viral suppression <1000 copies/mL. Cox proportional hazards models were used to calculate adjusted hazard ratios (aHRs) for associations with incident infections.

**Results:**

Between March 2013 and February 2019, 2123 participants were enrolled with median age 23 (interquartile range 21 to 27) years. Of 1745 tested, 865 (49.6%) were living with HIV. HIV incidence was 11.6/100 person‐years [PY], including 23.1/100PY (95% CI 15.5 to 33.1) among participants aged 16 to 19 years and 23.8/100 PY (95% CI 13.6 to 39.1) among TGW. Compared to participants aged ≥25 years, those aged 16 to 19 years had decreased odds of prior HIV testing (aOR 0.40 [95% CI 0.11 to 0.92]), disclosing same‐sex sexual practices to healthcare workers (aOR 0.53 [95% CI 0.36 to 0.77]) and receiving HIV prevention information (aOR 0.60 [95% CI 0.41 to 0.87]). They had increased odds of avoiding healthcare (aOR 1.94 [95% CI 1.3 to 2.83]) and engaging in transactional sex (aOR 2.76 [95% CI 1.92 to 3.71]). Age 16 to 19 years was independently associated with increased incidence of HIV (aHR 4.09 [95% CI 2.33 to 7.49]), NG (aHR 3.91 [95% CI 1.90 to 8.11]) and CT (aHR 2.74 [95% CI 1.48 to 5.81]).

**Conclusions:**

Young MSM and TGW demonstrated decreased healthcare engagement and higher incidence of HIV and other STIs as compared to older participants in this Nigerian cohort. Interventions to address unique obstacles to healthcare engagement by adolescents and young adults are needed to curb the spread of HIV and other STIs among MSM and TGW in Nigeria.

## 1 INTRODUCTION

Men who have sex with men (MSM) and transgender women (TGW) are disproportionately impacted by HIV and other sexually transmitted infections (STIs) [1, 2]. Young adult MSM and TGW are more likely than their older counterparts to engage in transactional sex, have multiple sexual partners and have condomless sex with partners whose HIV status remains unknown [1]. They tend to have lower levels of education, decreased ability to negotiate condom use and increased susceptibility to financial coercion to engage in condomless sex [3, 4]. They also tend to have more sexual partners and membership in higher risk sexual networks [5].

Same‐sex sexual practices are discouraged by cultural norms in many parts of the world and, as a result, MSM and TGW are frequently subject to stigma in their communities and from healthcare providers [6, 7, 8]. The synergistic interaction of stigma‐related challenges and sexual practices contributes to a decrease in healthcare seeking behaviour and engagement and an increase in the burden of HIV and other STIs among young adult MSM and TGW [8, 9]. In the United States where same‐sex practices are not criminalized, HIV incidence among MSM aged <25 years was 2.5 times that of MSM aged ≥25 years [10]. This phenomenon of higher HIV incidence among young adult MSM has consistently been replicated in other parts of the world [11, 12].

In Nigeria, Africa’s most populous country, same‐sex sexual practices are not only stigmatized, but criminalized [10, 13]. Experienced and anticipated stigma, such as expected rejection by their communities, lead many Nigerian MSM and TGW to avoid seeking healthcare [8, 14]. HIV screening is substantially less common among Nigerian MSM and TGW aged ≤19 as compared to those 30 years or older despite prior studies showing that awareness of HIV status lowers the risk of acquisition and transmission [15]. Young adult Nigerian MSM and TGW demonstrate a higher prevalence of sexual risk practices increasing their vulnerability to HIV and STIs as compared to their older counterparts [16]. We have previously demonstrated exceptionally high HIV incidence among Nigerian MSM and TGW, particularly those under 19 years of age [17].

The present analyses build upon this prior work to characterize age‐related determinants of healthcare engagement that may intersect with HIV acquisition risk. We explored healthcare‐seeking behaviour, HIV prevention practices, HIV care continuum outcomes and incidence of HIV, *Neisseria gonorrhoeae* (NG) and *Chlamydia trachomatis* (CT) infections in an updated data set with a focus on associations between age and each of these outcomes among MSM and TGW in Nigeria.

## 2 METHODS

### 2.1 Study design and population

This was a secondary data analysis of data collected in the TRUST/RV368 study of MSM and TGW in Nigeria. Participants were prospectively recruited into a combination HIV prevention and treatment study using respondent driven sampling (RDS) at two clinics in Abuja and Lagos as previously described [18, 19, 20].

The study was conducted in collaboration with local non‐governmental organizations at facilities tailored to the needs of sexual and gender minorities. Providers were sensitized to the social, legal and sexual health needs of MSM and TGW. Staff included members of the MSM and TGW communities. Services provided at the clinics included education about safer sex practices [21], distribution of condoms and condom compatible lubricants [22], as well as diagnosis and treatment of HIV and other STIs [23, 24, 25, 26].

The study was approved by the University of Maryland Baltimore IRB, Baltimore, MD, USA; the Federal Capital Territory Health Research Ethics Committee, Abuja, Nigeria; Ministry of Defense Health Research Ethics Committee, Abuja, Nigeria; and Walter Reed Army Institute of Research IRB, Silver Spring, MD, USA.

### 2.2 Data collection

Face‐to‐face interviews using structured questionnaires were administered to study participants at enrolment (spread across two visits spaced two weeks apart) and every three months thereafter for up to 18 months. Among other topics, the questionnaires captured data on demographic characteristics, sexual practices, healthcare‐seeking behaviours, disclosure of HIV status and receipt of HIV prevention education. Participants were also asked whether they were currently experiencing potential STI symptoms including penile or rectal discharge, painful urination, genital and ulcer/sore/lesion/rash.

Clinical data and specimens such as blood, rectal swabs and urine samples were collected at enrolment and each follow‐up visit. Blood samples were tested in real‐time for HIV using rapid HIV antibody tests following the parallel testing algorithm for at‐risk participants according to national guidelines in Nigeria with determine, Uni‐gold and HIV1/2 Stat Pak as a tie‐breaker for discrepant results [27]. For participants who were at risk for HIV, HIV testing continued every three months. Among people living with HIV (PLWH), plasma HIV RNA was quantified every three months (Abuja) or six months (Lagos) using COBAS TaqMan HIV‐1 Test (Roche Molecular Diagnostics, Pleasanton, CA, USA). At every visit, urine and rectal swabs were tested for NG and CT using the Aptima Combo 2 assay (Gen‐Probe, San Diego, CA, USA). All participants who tested positive for any infection were offered treatment according to national guidelines [28]. Partner tracing was not conducted as part of the study, but participants were encouraged to bring sexual partners to the clinic for STI diagnostics and treatment.

### 2.3 Definition of variables

HIV care continuum outcome variables included lifetime history of ever having been tested for HIV prior to enrolment, ART initiation at enrolment and viral suppression at enrolment. Participants who initiated ART and had HIV RNA < 1000 copies/mL at six‐months after ART initiation were considered virally suppressed, consistent with WHO Health [29]. Other outcomes included disclosure of HIV status and same‐sex sexual practices to health care workers, avoidance of seeking healthcare, participation in HIV prevention meetings, receipt of HIV prevention education and sex in exchange for money.

Incidence of HIV, rectal NG and CT infections were evaluated among participants who did not have each of these infections at enrolment. We chose to focus on rectal NG and CT for these analyses because we have previously reported that urogenital STIs are uncommon among Nigerian MSM and TGW [23, 25]. Only the first occurrence of NG and CT were included in these analyses. The main exposure of interest for all analyses was participant age at enrolment categorized as 16 to 19, 20 to 24 and ≥25 years. Age categories were chosen with a focus on the youngest participants in the cohort – those in their teenage years – with the remaining older participants divided into two groups of roughly equal size. Categorizing ≤19 as younger is consistent with our previous work [15]. All participants in the cohort were birth males and reported sex with men. We categorized participants as cisgender MSM, TGW, or other based on their self‐reported gender identity of “man,” “woman” or “other.” Network size was defined as the number of MSM and TGW the participant knew and/or had seen or communicated with in the past six months. Network density was calculated as the total number of actual connections divided by the total number of potential connections, with higher proportions indicating larger network densities [30]. Each study participant was asked to report up to 5 of his most recent MSM/TGW has interacted with within the past six months. Potential connections were then computed based on the number of MSM/TGW participant has interacted with. Network size was roughly categorized into four quartiles consistent to our previous work [15]. Social support was defined as the extent of assistance an individual was likely to receive from friends in times of need, based on responses to five different social support questions, each measured using a Likert scale ranging from (0) strongly disagree to (3) strongly agree. Scores were summed (score range: 0 to 15) and higher scores indicated stronger social support. For analyses, social support scores were dichotomized at the median in our study population.

### 2.4 Statistical analyses

Frequencies of categorical variables were calculated as the proportion of participants. Chi‐square tests were used to compare participants in relation to lifetime history of HIV testing, ART initiation and viral suppression. Logistic regression models were used to calculate odds ratios (ORs) and 95% confidence intervals (CIs) for age and other factors such as prior HIV testing, ART initiation, viral suppression, exposure to HIV education, disclosure of same‐sex sexual practices, healthcare‐seeking behaviours and transactional sex. Poisson regression models were used to compute incidence rates and 95% CIs of HIV, NG and CT infections. Cox proportional hazards models were used to calculate hazard ratios (HRs) associated with disease incidence. Person time was computed from the time of enrolment up to incident infection or study completion. Individuals with no incident infection were censored at their last study visit. In the primary analyses, cisgender MSM and TGW were pooled. Then, to explore whether the effect of age on the incidence of STIs was modified by HIV status or gender identity, analyses were stratified. Complete‐case analyses were conducted, excluding participants with missing data, except for analyses of HIV testing and linkage to care in which missing was recorded as not tested or not linked respectively. Enrolment data were used in cross‐sectional analyses to assess factors associated with prior HIV testing, ART initiation, exposure to HIV education, disclosure of same‐sex sexual practices, healthcare‐seeking behaviours and transactional sex. Longitudinal data were used to assess factors associated with incidence of HIV, NG and CT infections in participants who did not have each infection already at enrolment. Multivariable models were adjusted for gender identity, network size, network density and social support. These variables were adjusted because of their plausibility in relation to the outcomes explored. Statistical analyses were conducted using SAS version 9.3 (SAS Institute Inc., Cary, NC, USA).

## 3 RESULTS

### 3.1 Study population

**Figure 1:**
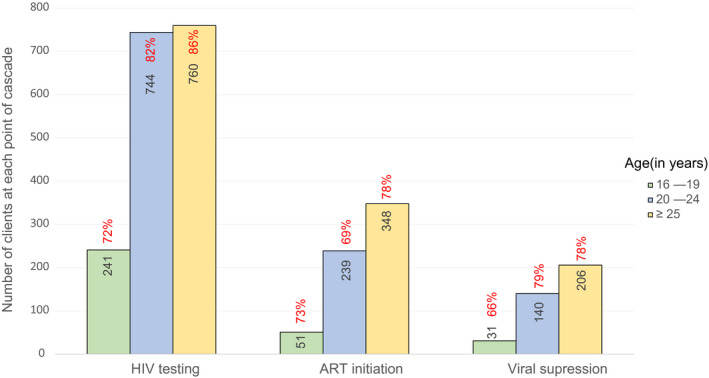
HIV care continuum outcomes. Comparison between younger and older MSM in Abuja and Lagos, Nigeria, 2013 to 2019. ART, antiretroviral therapy; MSM, men who have sex with men.

Between March 2013 and February 2019, a total of 2123 MSM and TGW were enrolled in TRUST/RV368, including 336 (15.8%) aged 16 to 19 years, 905 (42.6%) 20 to 24 years and 882 (41.5%) 25 years or older. Their median age was 23 (interquartile range 21 to 27) years. Among 2108 participants with self‐reported gender, 1697 (80.5%) were cisgender MSM, 234 (11.1%) were TGW and 177 (8.4%) reported other gender identities, such as non‐binary, gender neutral or gender fluid. Among 1745 participants who were tested for HIV upon enrolment, 865 (49.6%) were living with HIV. The prevalence of HIV was significantly higher among TGW compared to cisgender MSM (59.5% vs. 47.6%, *p* = 0.003). Proportion of missing data was in the range 0.7% to 26% for different outcome variables. Table 1 presents the distribution of covariates stratified by age groups. Table 2 presents outcome variables including HIV care continuum, markers of healthcare engagement and incident outcomes stratified by age group. Figure 1 presents HIV care continuum outcomes.

**Table 1:** Distribution of covariates and other characteristics: comparison between younger and older MSM in Abuja and Lagos, Nigeria, 2013 to 2019

Characteristics	16 to 19 years old	20 to 24 years old	≥25 years old	*p*‐value
	N = 336	N = 905	N = 882	
	n (%)	n (%)	n (%)	
Cis gender				
Cisgender MSM	266 (79.2)	711 (78.6)	720 (81.6)	
TGW	44 (13.1)	108 (11.9)	82 (9.3)	0.210
Other	26 (7.7)	80 (8.8)	72 (8.2)	
Missing	0 (0.0)	6 (0.7)	8 (0.9)	
Network density				
<50%	69 (20.5)	244 (27.0)	289 (32.8)	
≥50%	132 (39.3)	355 (39.2)	342 (38.8)	<.001
Missing	135 (40.2)	306 (33.8)	241 (28.5)	
Network size				
1 to 10	220 (65.5)	611 (67.5)	592 (67.1)	
11 to 20	47 (14.0)	131 (14.5)	132 (15.0)	
21 to 30	26 (7.7)	57 (6.3)	45 (5.1)	0.669
31+	43 (12.8)	102 (11.3)	110 (12.5)	
Missing	0 (0.0)	4 (0.4)	3 (0.3)	
Social support				
<Median	42 (12.5)	118 (20.8)	286 (32.4)	
≥Median	72 (21.4)	213 (23.5)	233 (26.4)	<.001
Missing	222 (66.1)	504 (55.7)	363 (41.6)	
Site				
Abuja	209 (62.2)	571 (63.1)	672 (76.2)	<.001
Lagos	127 (37.8)	334 (36.9)	210 (23.8)	

MSM, Men who have sex with men; n, number of study participants with a given characteristic; N, total number of study participants in that age category; TGW, transgender women.

**Table 2:** HIV care continuum outcomes, sti and health seeking behavior characteristics: comparison between younger and older MSM in Abuja and Lagos, Nigeria, 2013 to 2019

Characteristics	16 to 19 years old		20 to 24 years old		≥25 years old	
	N	n (%)	N	n (%)	N	n (%)
Tested for HIV	336	241 (71.7)	905	744 (82.2)	882	760 (86.2)
Initiated ART	70	51 (72.9)	349	239 (68.5)	446	348 (78.0)
Virally suppressed	47	31 (66.0)	178	140 (78.7)	263	206 (78.3)
Avoided seeking healthcare	336	82 (24.4)	902	196 (21.7)	874	179 (20.5)
Disclosed sex with men to healthcare worker	333	60 (18.0)	901	275 (30.5)	875	330 (37.7)
Disclosed HIV status to healthcare worker	36	29 (80.6)	187	152 (81.3)	262	218 (83.2)
Participated in HIV prevention meetings	197	45 (22.8)	609	186 (30.5)	660	191 (28.9)
Received information about HIV	197	146 (74.1)	610	483 (79.2)	663	548 (82.7)
Having anal sex in exchange for money	327	199 (60.9)	878	433 (49.3)	848	288 (34.0)
Cumulative HIV incidence	214	27 (12.6)	491	43 (8.7)	390	19 (4.9)
Cumulative Gonorrhoea incidence	122	28 (23.0)	453	101(22.3)	538	89 (16.5)
Cumulative Chlamydia incidence	120	33 (27.5)	473	110 (23.3)	548	105 (19.2)

ART, antiretroviral therapy; HIV, human immunodeficiency viruses; MSM, men who have sex with men; n, number of study participants with a given characteristic; N, total number of study participants in that age category; STI, sexually transmitted infections.

### 3.2 Prevalence of HIV and anorectal STIs

At enrolment, 865 participants were PLWH, of whom 380 (43.9%) were newly diagnosed during study enrolment. Among 1539 participants tested for NG, 311 (20.2%) had prevalent infection at enrolment and among 1537 tested for CT, 208 (13.5%) had prevalent infection.

### 3.3 HIV care continuum and other markers of healthcare engagement

Analyses of HIV care continuum and other markers of healthcare engagement (Table 3) showed that, compared to participants aged ≥ 25 years, those 16 to 19 years had decreased odds of prior HIV testing (adjusted odds ratios (aORs) = 0.40; 95% CI, 0.11 to 0.92), disclosing that they have sex with men to healthcare workers (aOR = 0.53; 95% CI, 0.36 to 0.77), and receiving information about HIV prevention (aOR = 0.60; 95% CI, 0.41 to 0.87). In comparison to older participants, those who were between 20 and 24 years old also had decreased odds of disclosing that they have sex with men to healthcare workers at enrolment (aOR = 0.80; 95% CI, 0.64 to 1.00) and for those living with HIV, had decreased odds of initiating ART (aOR = 0.60; 95% CI, 0.44 to 0.85). Young participants, had increased odds of having sex in exchange for money (16 to 19 vs. ≥ 25 aOR = 2.67; 95% CI, 1.92 to 3.71 and 20 to 24 vs. ≥ 25 aOR = 1.68; 95% CI, 1.34 to 2.10).

**Table 3:** Association between health seeking behaviour, HIV prevention characteristics and age: comparison between younger and older MSM/TGW in Abuja and Lagos, Nigeria, 2013 to 2019

Characteristics	16 to 19 years old	20 to 24 years old	≥25 years old
	aOR (95% CI)	aOR (95% CI)	aOR
Tested for HIV	0.40 (0.11 to 0.92)	0.50 (0.28 to 1.02)	Ref
Initiated ART	0.75 (0.43 to 1.38)	0.60 (0.44 to 0.85)	Ref
Viral suppression	0.82 (0.75 to 1.05)	1.10 (0.91 to 1.10)	Ref
Avoided seeking healthcare	1.94 (1.43 to 2.83)	1.11 (0.88 to 1.48)	Ref
Disclosed MSM status to healthcare	0.53 (0.36 to 0.77)	0.80 (0.64 to 1.00)	Ref
Disclosed HIV status to healthcare	0.84 (0.35 to 2.02)	0.90 (0.55 to 1.48)	Ref
Participated in HIV prevention meetings	0.92 (0.42 to 2.03)	1.36 (0.90 to 2.04)	Ref
Received information about HIV prevention	0.60 (0.41 to 0.87)	0.82 (0.46 to 1.48)	Ref
Having sex in exchange for money	2.67 (1.92 to 3.71)	1.68 (1.34 to 2.10)	Ref

aOR, adjusted odds ratio; ART, antiretroviral therapy; CI, confidence Intervals; HIV, human immunodeficiency viruses; MSM, men who have sex with men; n, number of study participants with a given characteristic; N, total number of study participants in that age category; TGW, transgender women.

### 3.4 Incident HIV and other STIs

The incidence of HIV per 100 person years among individuals aged 16 to 19 years was 23.1 (95% CI, 15.5 to 33.1). The incidence of HIV for those aged 20 to 24 and ≥25 years was 13.6 (95% CI, 9.9 to 18.1) and 5.8 (95% CI, 3.6 to 8.8) respectively. The incidence of anorectal NG per 100 person years among individuals aged 16 to 19, 20 to 24 and ≥25 years was 37.2 (95% CI, 25.2 to 53.1), 28.6 (95% CI, 23.4 to 34.6) and 15.8 (95% CI 12.8 to 19.3) respectively. The incidence of CT per 100 person years among individuals aged 16 to 19, 20 to 24 and ≥25 years was 41.1 (95% CI, 28.8 to 57.0), 26.9 (95% CI, 22.2 to 32.3) and 18.5 (95% CI, 15.2 to 22.3) respectively. Of 248 cumulative incident NG and CT infections, 201 (81.0%) were asymptomatic.

Compared to older participants, young participants had increased hazards of incident HIV, (adjusted hazard ratios (aHR) = 4.09; 95% CI, 2.33 to 7.47), NG (aHR = 3.91; 95% CI, 1.90 to 8.11) and CT (aHR = 2.74; 95% CI, 1.48 to 5.81), infections after adjusting for gender, network size, network density and social support (Table 4). As compared to cisgender MSM, TGW had a higher hazards of incident HIV, (aHR = 2.00; 95% CI, 1.09 to 3.61), NG (aHR = 1.71; 95% CI, 1.00 to 2.79) and CT (aHR = 1.42; 95% CI, 1.00 to 2.01). Analyses stratified by HIV (Table 5) showed that age, 16 to 19 vs.> 25 years (aHR = 2.00; 95% CI: 1.19 to 3.01) and 20 to 24 vs. ≥25 years (aHR = 3.88; 95% CI: 1.91 to 3.61), was significantly associated with an increased hazards of incident NG among participants living with HIV. Similarly, those who were 16 to 19 vs. ≥25 years (aHR = 1.73; 95% CI: 1.22 to 2.59) and 20 to 24 vs. ≥25 years (aHR = 2.68; 95% CI: 1.30 to 5.72) had an increased hazard of incident CT among participants living with HIV. There were no statistically significant association between network density, network size, social support and incidence of HIV or other STIs.

**Table 4:** Factors associated with incident HIV/STI among MSM and TGW in Abuja and Lagos, Nigeria, 2013 to 2019

Characteristic	HIV (N = 880)			Gonorrhoea (N = 1141)			Chlamydia (N = 1113)		
	Cases/person years	Incidence /100 person years	aHR (95% CI)	Cases/person years	Incidence /100 person years	aHR (95% CI)	Cases/person years	Incidence /100 person years	HR (95% CI)
Age									
≥25	19/330.0	5.8	Ref	89/563.7	15.8	Ref	105/567.6	18.5	Ref
20 to 24	43/317.0	13.6	**2.41 (1.39 to 4.11)**	101/353.7	28.6	**1.86 (1.36 to 2.43)**	110/408.5	26.9	**1.67 (1.18 to 2.60)**
16 to 19	27/117.0	23.1	**4.09 (2.33 to 7.47)**	28/75.2	37.2	**3.91 (1.90 to 8.11)**	33/80.3	41.1	**2.74 (1.48 to 5.81)**
Cisgender									
Cisgender MSM	65/631.2	10.3	Ref	166/813.0	20.4	Ref	187/854.2	21.9	Ref
TGW	14/58.7	23.8	**2.00 (1.09 to 3.61)**	31/92.7	33.4	**1.72 (1.00 to 2.79)**	36/106.1	33.9	**1.42 (1.00 to 2.01)**
Other	8/49.9	16.0	1.39 (0.64 to 2.88)	18/62.3	28.9	1.69 (0.92 to 3.18)	19/70.8	26.8	1.28 (0.78 to 1.99)
Network density									
<50%	45/337.0	13.4	Ref	123/537.5	22.9	Ref	111/482.6	23.0	Ref
≥50%	40/352.7	11.3	0.82 (0.49 to 1.17)	95/454.0	20.9	0.71 (0.49 to 1.13)	132/546.6	24.0	1.00 (0.71 to 1.53)
Network size									
1 to 10	61/478.0	12.8	Ref	132/577.2	22.9	Ref	136/621.2	21.9	Ref
11 to 20	7/115.1	6.1	0.52 (0.21 to 1.14)	33/176.0	18.8	0.78 (0.54 to 1.22)	50/171.8	29.1	**1.32 (1.01 to 1.78)**
21 to 30	6/68.4	8.8	0.58 (0.24 to 1.28)	14/68.4	20.5	0.84 (0.50 to 1.51)	21/80.7	26.0	1.11 (0.72 to 1.84)
31+	14/85.4	16.4	1.32 (0.68 to 2.32)	39/167.3	23.3	0.93 (0.71 to 1.46)	41/179.7	22.8	1.00 (0.68 to 1.41)
Social support									
<Median	40/354.5	11.3	Ref	98/438.5	22.3	Ref	137/582.3	23.5	Ref
≥Median	48/366.7	13.1	1.09 (0.66 to 1.82)	117/527.0	22.2	1.18 (0.81 to 1.88)	111/472.9	23.4	0.78 (0.51 to 1.23)

CI, confidence intervals; HIV, human immunodeficiency viruses; HR, hazard ratio; MSM, men who have sex with men; STI, sexually transmitted infections; TGW, transgender women.

**Table 5:** Factors associated with incident GC and CT among MSM and TGW in Abuja and Lagos, Nigeria, 2013 to 2019

Characteristic	Gonorrhoea				Chlamydia			
	HIV‐infected		HIV‐uninfected		HIV‐infected		HIV‐uninfected	
	Cases/person years	aHR (95% CI)	Cases/person years	aHR (95% CI)	Cases/person years	aHR (95% CI)	Cases/person years	aHR (95% CI)
Age								
≥25	70/369.5	Ref	19/193.9	Ref	79/382.5	Ref	26/184.9	Ref
20 to 24	81/224.4	2.00 (1.29 to 3.01)	20/129.0	1.51 (0.78 to 3.02)	87/270.6	1.73 (1.22 to 2.59)	23/137.5	1.18 (0.72 to 2.12)
16 to 19	22/40.4	3.88 (1.91 to 7.99)	6/34.9	2.01 (0.83 to 4.99)	23/50.0	2.68 (1.30 to 5.72)	10/30.3	2.89 (1.52 to 6.14)
Network density								
<50%	71/228.5	Ref	30/213.5	Ref	71/266.0	Ref	40/216.6	Ref
≥50%	85/322.1	0.99 (0.71 to 1.43)	32/213.9	1.22 (0.72 to 1.89)	95/348.3	0.99 (0.72 to 1.53)	37/197.9	1.14 (0.67 to 1.71)
Network size								
1 to 10	88/309.2	Ref	44/268.4	Ref	93/344.8	Ref	43/276.2	Ref
11 to 20	24/106.9	0.92 (0.63 to 1.49)	9/69.0	0.74 (0.29 to 1.52)	33/105.8	1.33 (0.89 to 2.01)	17/65.9	1.42 (0.78 to 2.62)
21 to 30	11/40.5	1.01 (0.48 to 1.87)	3/27.8	0.63 (0.21 to 2.24)	13/48.4	0.91 (0.48 to 1.70)	8/32.1	1.51 (0.72 to 3.32)
31+	33/105.9	1.12 (0.67 to 1.73)	6/61.3	0.49 (0.24 to 1.29)	30/127.2	0.82 (0.58 to 1.31)	11/52.4	1.11 (0.63 to 2.28)
Social support								
<Median	93/323.9	Ref	27/210.0	Ref	97/357.8	Ref	40/203.0	Ref

aHR, adjusted Hazard ratio; CI, confidence intervals; CT, *Chlamydia trachomatis*; GC, *Neisseria gonorrhoeae*; HIV, human immunodeficiency viruses; TGW, transgender women.

The effect of age on incident HIV, NG and CT infections was modified by whether a participant identified as cisgender MSM or TGW. Cisgender MSM who were 16 to 19 vs. ≥25 years (aHR = 4.70; 95% CI: 2.28 to 9.51) and 20 to 24 vs. ≥25 years (aHR = 2.83; 95% CI: 1.48 to 5.32) had increased hazards of incident HIV (Table S1). Cisgender MSM who were 16 to 19 vs. ≥25 years (aHR = 2.70; 95% CI: 1.65 to 4.41) and 20 to 24 vs. ≥25 years (aHR = 1.81; 95% CI: 1.30 to 2.51) had increased hazards of incident NG (Table S2). Cisgender MSM who were 16 to 19 vs. ≥25 years (aHR = 1.63; 95% CI: 1.66 to 4.16) and 20 to 24 vs. ≥25 years (aHR = 1.60; 95% CI: 1.17 to 2.18) had increased hazards of incident CT (Table S3). There were no statistically significant associations between age and incident HIV, NG or CT among TGW.

## 4 DISCUSSION

The incidence of HIV and other STIs was higher among cisgender MSM and TGW youth aged 24 and younger as compared to older cisgender MSM and TGW. The incidence of HIV and other STIs was also higher among Nigerian TGW compared to cisgender MSM. As previously reported, TGW who have sex with men predominantly practice receptive anal sex that carried an increased risk of HIV and other STI acquisition as compared to other sex acts [31]. High burdens of bacterial STIs have been reported among TGW, which also increase risk of incident HIV infection [32, 33]. High incidence of HIV has similarly been reported among TGW in Kenya [34]. We have also shown that the incidence rates of HIV and other STIs were significantly higher among cisgender MSM and TGW youth compared to older cisgender MSM and TGW in Nigeria. Globally, young populations may face many developmental, psychological and social factors that predispose them to an increased risk of STIs [35]. These may include lack of knowledge about HIV risk, negative or complacent attitudes towards safer sex, disinformation provided by peers on safer sexual behaviours, not having experienced the severity of disease at the onset of the illness and perception of HIV as a chronic disease, which may lead to underestimation of personal risk [18, 36, 37]. Our findings also showed cisgender MSM and TGW youth were less likely to participate in HIV prevention meetings or receive information about HIV prevention and therefore less likely to be knowledgeable of HIV prevention strategies. High HIV incidence among cisgender MSM and TGW youth underscores the need for biomedical interventions to prevent HIV, such as pre‐exposure prophylaxis (PrEP), and the need for innovative strategies that improve uptake and adherence to these interventions. Potential barriers to PrEP use by young MSM include aversion to daily pills and frequent clinic visits [38].

Regular HIV testing is critical for ensuring that cisgender MSM and TGW youth are linked to appropriate HIV prevention and treatment services. Frequent testing and treatment are also beneficial in diagnosing other STIs and reducing undiagnosed HIV infection [39, 40, 41]. In these analyses, we noted young cisgender MSM and TGW reported avoidance of healthcare. Prior studies have shown that individuals at risk for HIV fear rejection and additional stigma if they acquire HIV, hindering them from accessing HIV testing [42]. Qualitative literature has shown that young MSM experience fear of HIV testing results, rejection and unfriendly testing environments [43]. Prior studies also indicate that PLWH may delay or not access care in order to avoid anticipated rejection by providers, families and the general public [15, 16]. Low levels of testing, particularly among MSM and TGW youth, could hinder implementation of HIV prevention strategies such as PrEP.

Over half of the cisgender MSM and TGW youth in this cohort reported having sex in exchange for money. A prior study from Nigeria also showed that transactional sex was most common among MSM aged between 15 and 19 years [16]. We have previously reported that sex‐selling was independently associated with decreased uptake of healthcare services in our cohort [44]. Researchers have noted that transactional sex is more prevalent for those who are socio‐economically strained and increases the rates of HIV and other STIs [45]. Compared to older MSM, young MSM are more likely to be economically disadvantaged and hence engage in sex‐selling. It is also important to note that selling sex is associated with high‐risk behaviours such as condomless sex [46, 47]. Higher incidence of HIV and other STIs among cisgender MSM and TGW youth in this cohort could also be due to the high proportion of engagement in transactional sex and mediated by condomless sex.

These analyses included a large sample from a well‐characterized cohort of cisgender MSM and TGW in combination with evaluation of HIV care continuum, markers of healthcare engagement and incident outcomes, improving upon earlier cross‐sectional and cohort studies with smaller sample sizes evaluating age‐related determinants of healthcare needs and engagement [48, 49]. A limitation of this study was loss to follow‐up which contributed to a high amount of missing data, ranging from 0.7% to 26% for different outcome variables. Also, our use of a complete‐case strategy for analyses resulted in different sample sizes and denominators for most analyses and may have introduced some bias, but enabled inclusion of diverse parameters without making any assumptions for imputation of missing values. Stigma, mental health and substance use were not used in the secondary analysis and this limited our ability to describe their association with incidence of HIV and other STIs. However, our previous work showed that higher stigma scores were associated with increased prevalence of HIV and STIs [6]. In addition, the small TGW sample size diminished power to detect significant differences in HIV or STI incidence by age group, though the observed rates were indeed higher among younger age groups among TGW. RDS weights were not included in our models based on our prior work showing that equilibrium for our HIV incidence analysis was reached after recruitment of 100 to 300 individuals [17]. HIV testing was assessed using an ever measure, which limited our ability to understand with certainty if young individuals were less likely than older ones to test for HIV. Furthermore, differences in the lower age limit (16 for Abuja and 18 for Lagos) for recruitment may have introduced bias in our estimates of age‐related associations. To distinguish new incident infections from unresolved infections, we excluded prevalent anorectal STI and considered only the first incident infection in all incidence analyses.

## 5 CONCLUSIONS

The incidence of HIV and other STIs among Nigerian cisgender MSM and TGW youth participants was remarkably high in this cohort. Developmental and social transitions that occur during this period of life limit the exposure of youth to HIV and STI prevention strategies. This calls for focused and comprehensive prevention messaging as well as care facilities that specialize in adolescent care to better engage cisgender MSM and TGW youth. In many settings, exposure to sexual health and HIV prevention education doesn’t begin until after individuals reach 18 years of age, however, sexual debut can be much earlier. Early engagement to address the healthcare needs of youth at risk for HIV and other STIs is critical in order to achieve an AIDS free generation.

## COMPETING INTERESTS

The authors declare no conflict of interest.

## AUTHORS’ CONTRIBUTIONS

HOR designed this analysis and authored the first draft of the manuscript. BOK, UO, ES, CE and AK oversaw the collection of clinical data. TAC, RGN, NN, SA, SDB and MEC, contributed to the design of the analysis and assisted in the interpretation of results. All authors reviewed this manuscript, provided feedback and approved of the manuscript in its final form.

## Supporting information


**Table S1. **Association between incident HIV and age: comparison between MSM and TGW in Abuja and Lagos, Nigeria, 2013 to 2019
**Table S2.** Association between incident gonorrhea and age: comparison between MSM and TGW in Abuja and Lagos, Nigeria, 2013 to 2019
**Table S3. **Association between incident chlamydia and age: comparison between MSM and TGW in Abuja and Lagos, Nigeria, 2013 to 2019
