# Supplementary material for: Association of age with healthcare needs and engagement among Nigerian men who have sex with men and transgender women: cross‐sectional and longitudinal analyses from an observational cohort
Source: J Int AIDS Soc. 2020 Oct 1;23(Suppl 6):e25599. doi: 10.1002/jia2.25599 (PMC7527771; doi:10.1002/jia2.25599)
Supplement: Supplementary file 1 — Table S1. Association between incident HIV and age: comparison between MSM and TGW in Abuja and Lagos, Nigeria, 2013 to 2019 Table S2. Association between incident gonorrhea and age: comparison between MSM and TGW in Abuja and Lagos, Nigeria, 2013 to 2019 Table S3. Association between incident chlamydia and age: comparison between MSM and TGW in Abuja and Lagos, Nigeria, 2013 to 2019 [file JIA2-23-e25599-s001.docx]

| Supplementary Table 1: Association Between incident HIV and Age: Comparison Between MSM and TGW in Abuja and Lagos, Nigeria, 2013 – 2019. | | | | | | | | | |
| --- | --- | --- | --- | --- | --- | --- | --- | --- | --- |
|  |  |  |  |  |  |  |  |  |  |
| Characteristic | MSM (N= 724) | | | TGW (N= 76) | | | Other (N = 72) | | |
|  | Cases/ Person years | Incidence /100 person years | HR (95% CI) | Cases/ Person years | Incidence /100 person years | HR (95% CI) | Cases/ Person years | Incidence /100 person years | HR (95% CI) |
|  |  |  |  |  |  |  |  |  |  |
|  |  |  |  |  |  |  |  |  |  |
|  |  |  |  |  |  |  |  |  |  |
| Age |  |  |  |  |  |  |  |  |  |
| ≥ 25 | 13/282.6 | 4.6 | Ref | 1/18.2 | 5.5 | Ref | 4/18.5 | 21.6 | Ref |
| 20 – 24 | 33/257.8 | 12.8 | 2.83(1.48– 5.32) | 7/26.9 | 26.0 | 5.52(0.64 – 48.01) | 2/22.3 | 9.0 | 0.47(0.11 – 2.90) |
| 16 – 19 | 19/90.8 | 20.9 | 4.70(2.28 – 9.51) | 6/13.7 | 43.8 | 8.00(1.02 – 67.21) | 2/9.2 | 21.7 | 1.12(0.24 – 6.41) |
| Abbreviations: HR, Hazard ratio; CI, Confidence intervals; MSM, men who have sex with men; TGW, Transgender women; HIV, Human immunodeficiency viruses. | | | | | | | | | |
|  |  |  |  |  |  |  |  |  |  |
|  |  |  |  |  |  |  |  |  |  |

| Supplementary Table 2: Association Between incident Gonorrhea and Age: Comparison Between MSM and TGW in Abuja and Lagos, Nigeria, 2013 – 2019. | | | | | | | | | |
| --- | --- | --- | --- | --- | --- | --- | --- | --- | --- |
|  |  |  |  |  |  |  |  |  |  |
| Characteristic | MSM (N= 898) | | | TGW (N= 108) | | | Other (N = 98) | | |
|  | Cases/ Person years | Incidence /100 person years | HR (95% CI) | Cases/ Person years | Incidence /100 person years | HR (95% CI) | Cases/ Person years | Incidence /100 person years | HR (95% CI) |
|  |  |  |  |  |  |  |  |  |  |
|  |  |  |  |  |  |  |  |  |  |
|  |  |  |  |  |  |  |  |  |  |
| Age |  |  |  |  |  |  |  |  |  |
| ≥ 25 | 69/468.7 | 14.7 | Ref | 8/41.4 | 19.3 | Ref | 11/49.5 | 22.2 | Ref |
| 20 – 24 | 76/287.4 | 26.4 | 1.81(1.30 – 2.51) | 17/39.5 | 43.0 | 2.20(0.95 – 5.12) | 7/26.9 | 26.9 | 0.93(0.12 – 7.32) |
| 16 – 19 | 21/57.6 | 36.4 | 2.70(1.65 – 4.41) | 6/11.8 | 50.8 | 2.47(0.85 – 7.18) | 1/16.9 | 41.1 | 1.35(0.54 – 3.40) |
| Abbreviations: HR, Hazard ratio; CI, Confidence intervals; MSM, men who have sex with men; TGW, Transgender women | | | | | | | | | |
|  |  |  |  |  |  |  |  |  |  |
|  |  |  |  |  |  |  |  |  |  |

| Supplementary Table 3: Association Between incident Chlamydia and Age: Comparison Between MSM and TGW in Abuja and Lagos, Nigeria, 2013 – 2019. | | | | | | | | | |
| --- | --- | --- | --- | --- | --- | --- | --- | --- | --- |
|  |  |  |  |  |  |  |  |  |  |
| Characteristic | MSM (N= 917) | | | TGW (N= 114) | | | Other (N = 101) | | |
|  | Cases/ Person years | Incidence /100 person years | HR (95% CI) | Cases/ Person years | Incidence /100 person years | HR (95% CI) | Cases/ Person years | Incidence /100 person years | HR (95% CI) |
|  |  |  |  |  |  |  |  |  |  |
|  |  |  |  |  |  |  |  |  |  |
|  |  |  |  |  |  |  |  |  |  |
| Age |  |  |  |  |  |  |  |  |  |
| ≥ 25 | 78/468.1 | 16.7 | Ref | 13/42.7 | 30.4 | Ref | 14/51.8 | 27.0 | Ref |
| 20 – 24 | 85/327.4 | 26 | 1.60(1.17– 2.18) | 17/47.7 | 35.6 | 1.14(0.55 – 2.35) | 8/32.6 | 24.5 | 0.93(0.41 – 2.33) |
| 16 – 19 | 24/59.4 | 40.4 | 2.63(1.66 – 4.16) | 6/15.7 | 38.2 | 1.26(0.47 – 3.38) | 3/5.2 | 57.7 | 2.61(0.72 – 9.37) |
| Abbreviations: HR, Hazard ratio; CI, Confidence intervals; MSM, men who have sex with men; TGW, Transgender women | | | | | | | | | |
|  |  |  |  |  |  |  |  |  |  |
|  |  |  |  |  |  |  |  |  |  |
